# Seasonal and evolutionary patterns of planktonic Hikarchaeia reveal ecological niches of marine rare biosphere constituents

**DOI:** 10.1128/mbio.01753-26

**Published:** 2026-10-19

**Authors:** Fabian Wittmers, Nicole R. Dames, Michelle Michelsen, Rachele Spezzano, Jacqueline Comstock, Kristin Bergauer, Kevin Vergin, Shuting Liu, Rachel Parsons, Ben Temperton, Mak A. Saito, Craig A. Carlson, Alexandra Z. Worden

**Affiliations:** 1 Marine Biological Laboratory 42700, Woods Hole, Massachusetts, USA; 2 School of Biosciences, University of Exeter, Exeter, Devon, United Kingdom; 3 Department of Geophysical Sciences, University of Chicago, Chicago, Illinois, USA; 4 Department of Ecology, Evolution and Marine Biology and Marine Science Institute, University of California, Santa Barbara, California, USA; 5 GEOMAR Helmholtz Centre for Ocean Research Kiel, Kiel, Germany; 6 Microbial DNA Analytics, Phoenix, Oregon, USA; 7 Department of Environmental & Sustainability Sciences, Kean University, Union, New Jersey, USA; 8 Bermuda Institute of Ocean Sciences, School of Ocean Futures, Arizona State University 128687, St George’s, Bermuda; 9 Department of Marine Chemistry and Geochemistry, Woods Hole Oceanographic Institution 10627, Woods Hole, Massachusetts, USA; The University of Oklahoma, Norman, Oklahoma, USA

**Keywords:** archaeal evolution, Marine Group IV, ocean time series, BATS, Archaea, seasonality, Hikarchaeia

## Abstract

Members of Marine Group IV, now termed Hikarchaeia, hold an important transitional evolutionary position among Archaea but remain uncultivated, and their ecology remains uncharacterized. Over a 5-year period, we performed monthly profile sampling in the North Atlantic Subtropical Gyre and sequenced V4 16S rRNA gene amplicons, while also collecting additional size-fractionated and metagenomic samples. Hikarchaeal relative abundance annually peaked at ~1% of prokaryotic amplicons. Their relative abundances were significantly higher after deep winter mixing when thermal stratification isolated exported dissolved organic matter in the twilight zone (TZ), the boundary region between the epipelagic and mesopelagic. A diminutive 1.17 Mbp complete hikarchaeal genome was recovered by sequencing and co-assembling diel TZ metagenomes from 200 m during summer, where their relative abundances were highest. Phylogenomic reconstruction incorporating our new metagenome-assembled genome (MAG) and public MAGs established five clades. Clades I and III were in subtropical and tropical environments, with vertical partitioning: Clade I dominated the TZ, and Clade III detection initiated in the lower mesopelagic and extended into the bathypelagic. Clade V is polar-associated. Data sparsity makes Clade II and IV distributions unclear. All Hikarchaeia recovered thus far appear to be free-living aerobes capable of heterotrophy. Unlike MGI, MGII, and MGIII Archaea, and most marine pelagic bacteria, the Hikarchaeia encode bacterioruberin biosynthesis, except deep Clade III. Hikarchaeia also have nitrile hydratases alongside enzymes for carbon monoxide oxidation and menaquinone biosynthesis, suggesting previously unrecognized roles in nitrogen and carbon cycling. Collectively, our studies reveal niche partitioning of evolutionarily distinct uncultivated Hikarchaeia clades and patterning within the rare biosphere.

**IMPORTANCE:**

The ocean and its vertical water-column structure are changing with planetary warming; yet, there are many enigmatic microbes, especially those that inhabit the deep ocean. Here, we illuminate a group of uncultivated Archaea that were discovered over two decades ago but still lack clear distributions and habitat preferences. With the first complete hikarchaeal genome now in hand, we identified distinct lineages within the Hikarchaeia that occupy different marine niches. Strong reoccurring seasonal patterns were observed that closely followed the annual cycle of temperature-driven ocean stratification. Given rapid natural and anthropogenic perturbations at hand, establishing baselines for these Archaea is critical for improving our understanding and modeling of marine microbiomes and their biogeochemical functions.

## INTRODUCTION

Archaea are important members of ocean water-column microbial communities and harbor multiple evolutionarily distinct groups (1–4). Currently, there are 19 designated phyla, several of which contain pelagic marine groups, and many are still uncultivated (3–6). The pelagic lineages were initially designated “Marine Groups” (MG) I to IV at the time of their discoveries in environmental 16S rRNA clone libraries (4, 7–10). Although these lineages remain the key pelagic marine archaeal groups, they are now known to come from different phyla (1, 3), and naming is constantly evolving. MGI (class Thaumarchaeota or Nitrososphaeria) belongs to the phylum Thermoproteota, and several strains are now in culture (11–17). The others belong to the tentative superphylum Euryarchaeota, specifically MGII (Poseidoniales) and MGIII (6, 18–20), which are both orders within the class Poseidoniia (Thermoplasmatota phylum). Marine Group IV belongs to the phylum Halobacteriota (21–23), which is also in the tentative superphylum Euryarchaeota. MGII, MGIII, and MGIV remain uncultivated, limiting understanding of their biology and physiology. MGII appears ubiquitous in the ocean, with higher abundances in surface waters, especially in coastal environments (19, 24–26). Less is known about distributions of MGIII and especially MGIV, which are considered rare (1, 18, 27).

MGIV was first discovered at 3,000 m in the Antarctic Polar Front via 16S rRNA gene clone libraries (28). A MGIV sequence was next recovered from Arctic surface waters (~40–130 m) and near a 2,540 m Pacific volcano plume (28–30). These findings indicated that MGIV was deep-sea adapted or potentially a cold-water specialist. More recently, MGIV metagenome-assembled genomes (MAGs) were described from the tropical Pacific Ocean (5–120 m), Southern Ocean (790 m), and Red Sea (80 m) (23). While initially referred to as MGIV Archaea (30), and later as euryarchaeal MGIV, phylogenomic analyses then reclassified this lineage as the order UBA12382 (GTDB [31]) or the class Hikarchaeia (23).

Evolutionary studies incorporating the initial five MAGs representing Hikarchaeia focused on transitions in methanogen and halophile evolution, with varying interpretations depending on the analyses (21, 23). These studies utilized the two HQ-MAGs (HikBin1 and HikBin2) and three MQ-MAGs (HikBin3–5) available at the time for Hikarchaeia, inferring they are intermediates in the methanogen-to-halophile transition, which includes loss of genes required for methanogenesis and for dark carbon fixation through the Wood-Ljungdahl pathway. The MAG-based results also indicated that Hikarchaeia have minimally redundant, relatively small genomes with metabolisms indicative of an aerobic lifestyle and encode enzymes for utilizing organic substrates for heterotrophic growth (23). Finally, they encode all archaeal lipid biosynthesis proteins (32), as does MGI but not MGII and MGIII (31).

Archaea have been increasingly recognized in the surface ocean, especially MGII, and have long been known to contribute significantly below the photic zone, especially MGI (19, 33–35). Microbial activities and respiration in dark ocean layers, i.e., the mesopelagic (200–1,000 m) and bathypelagic (>1,000 m), influence the transfer efficiency of the biological carbon pump—by which phytoplankton biomass is exported out of sunlit surface waters (36–38). To date, however, little is known about the ecology and distributions of Hikarchaeia (3, 23). This is partially due to limitations in taxonomic resolution in field studies; for example, the highest archaeal abundances in mid-Atlantic Ocean qPCR data at North Pond were in the mesopelagic, whereas the low relative abundance archaeal group Halobacteria was enriched in bathypelagic amplicons (27). The latter may include contributions from Hikarchaeia (based on SILVA taxonomic placement within the Halobacteria), but so far, their specific contributions remain unclear. Furthermore, it has been proposed that HikBin1–5 represent only a portion of hikarchaeal diversity based on comparisons to 16S rRNA gene clone library data (23).

Here, we identify and link hikarchaeal features to seasonal biogeochemistry at the Bermuda Atlantic Time-series Study (BATS) site in the Sargasso Sea (38). To resolve depth-distribution patterns and potential ecological differentiation, we performed V4 16S rRNA gene amplicon sequencing for 607 profile samples primarily from 0 to 300 m, but with some reaching 1,000 m. We also leveraged V4 16S rRNA gene amplicon data from 396 samples from large-volume size-fractionated collections conducted within the same time period (39). After establishing relative abundance patterns of Hikarchaeia over the seasons, additional sampling was performed to pursue their distributions in the bathypelagic and generate metagenomic information for this uncultivated archaeal group. Subsequent phylogenomic analyses of the resulting complete MAG from 200 m at BATS, and other publicly available MAGs, established multiple unrecognized clades that have habitat specificities, based on available data. Functional analyses of their diminutive genomes revealed the presence of nitrile hydratase, an important enzyme in nitrogen cycling (40), and other novel features that are lacking in MGI, II, and III. Collectively, these studies provide new insights into adaptations, diversification, and distributions of Hikarchaeia.

## RESULTS AND DISCUSSION

**Fig 1:**
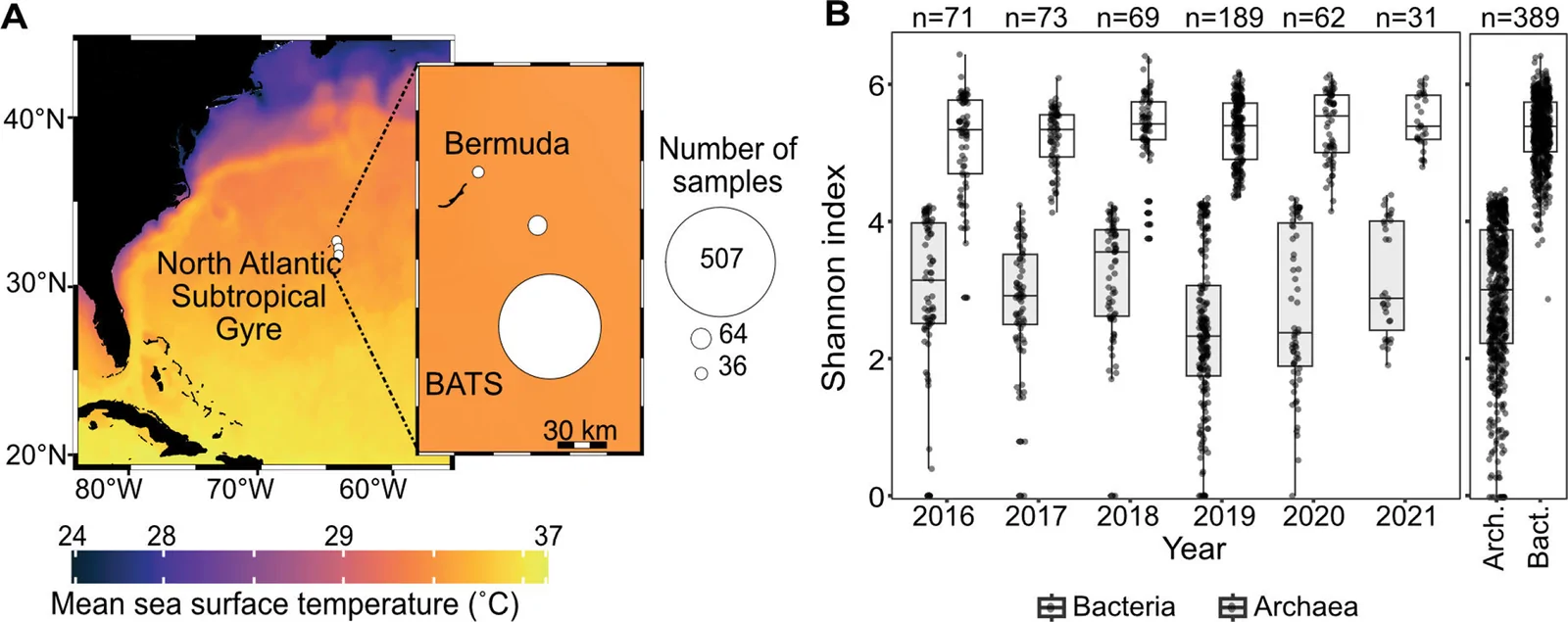
Microbial sampling and diversity in the oligotrophic subtropical Atlantic Ocean. (**A**) Location of sample collections at the BATS site and nearby collections used herein from 2016 to 2021 (white circles). Bubble sizes indicate the number of samples from each location, and bubbles are underlain by annual average night-time sea surface temperature (°C) at blended 5 km resolution. (**B**) Shannon diversity for the upper 300 m of the water column for each year sampled, partitioned between bacteria and archaea (left panel) and between 1 and 1,000 m for 2019–2021 (right panel). Note that diversity index values close to or at zero result from samples with low or zero archaeal amplicons.

Over a period of 5 years, spanning 2016 to 2021, 607 DNA samples were collected while performing monthly water-column profiles at BATS (Fig. 1A). Biomass was collected on a 0.22 µm filter, extracted, and the V4 region of the 16S rRNA gene was amplified and sequenced, resulting in a total of 6.03 × 10^7^ prokaryotic amplicons from the 602 samples that passed quality thresholds (Data set S1). Among these, 495 were from the upper 300 m of the water column, forming the set used to examine seasonal patterns. Others (107 samples) came from depths >300 m (500, 600, 800, and 1,000 m), where sampling was sparser. Archaea were detected in 99% of the samples, with four surface samples lacking archaeal amplicons. Diversity metrics indicated richness was similar for each of the years sampled, with archaeal values being consistently lower and more variable than bacterial richness in the upper 300 m, and likewise in years when profile sampling reached 1,000 m (Fig. 1B).

### Distributions of Hikarchaeia in the subtropical ocean

**Fig 2:**
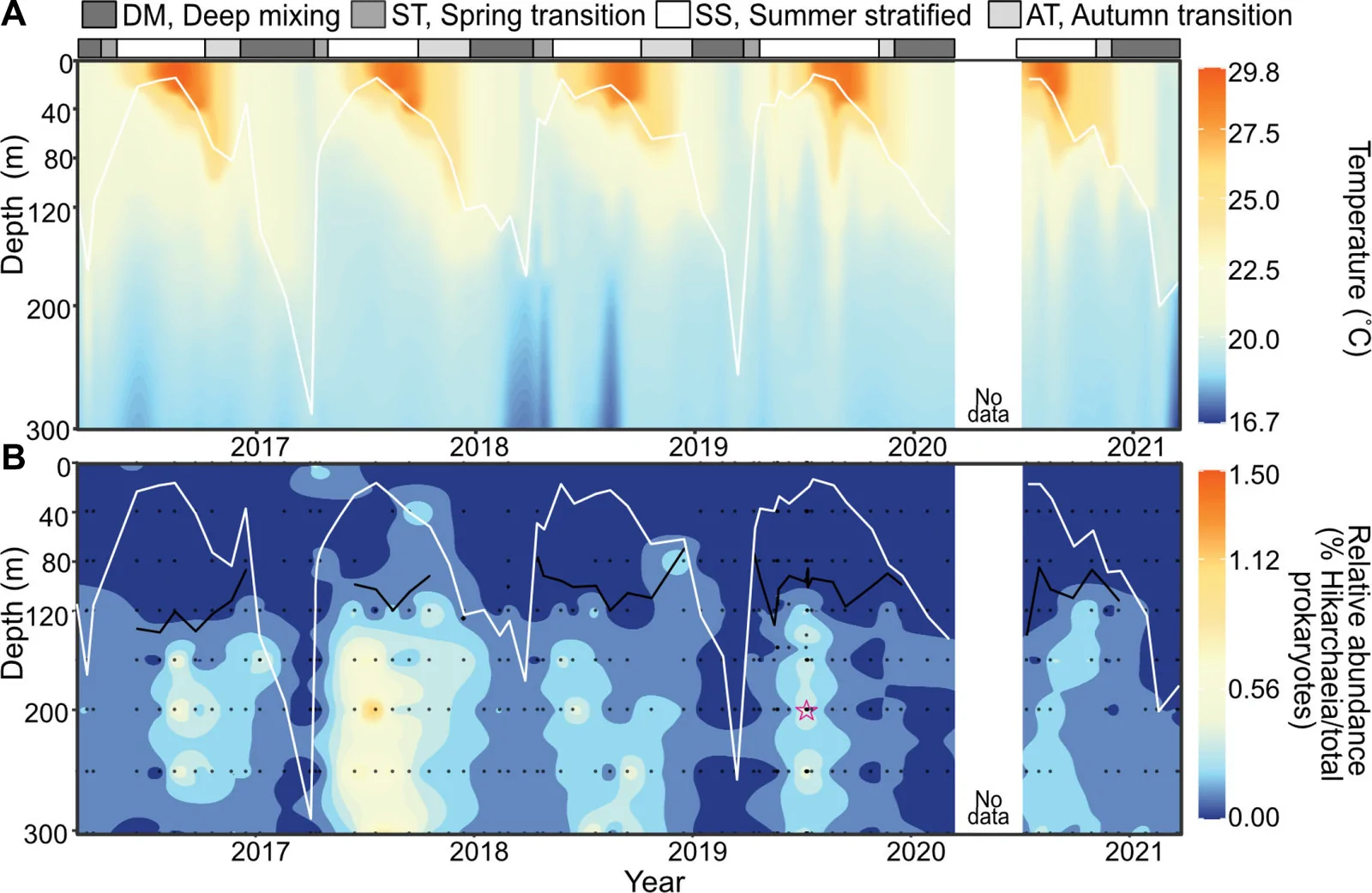
Seasonal dynamics and distribution of Hikarchaeia at BATS. (**A**) Temperature (°C) over the upper 300 m of the BATS water column based on conductivity-temperature-depth (CTD) data. Water-column stability periods (above plot) are based on physical characteristics of the water column. Computed seasonal mixed layer depths (white line) are indicated. (**B**) Relative amplicon abundance of Hikarchaeia out of total prokaryotes based on V4 16S rRNA gene data (Data set S1) with interpolation from discrete samples (black dots, *n* = 495). When present the DCM is indicated (black line). The star shows the depth and date of the six metagenomes used to assemble the HikBATS200m MAG (Data set S3).

Seasonal trends in temperature and other variables align with prior results from this oligotrophic open-ocean site (41, 42). Briefly, winter and spring deep convective mixing in the upper 300 m was followed by water-column stratification in summer and autumn, as manifested in temperature data (Fig. 2A), with the stratified period being considered representative of future oceans (43). A deep chlorophyll maximum (DCM) was present primarily during the summer stratified period and autumn transition, at depths between 84 and 139 m, similar to dynamics reported in reference 44. These features have been shown to shape other biotic and abiotic parameters at BATS. Specifically, the winter deep mixing causes export of surface-accumulated dissolved organic matter (DOM) from the photic zone to the twilight zone (TZ). Upon stratification, a significant proportion of exported DOM is trapped below the seasonal mixed layer in a region termed the twilight zone (41, 45). The twilight zone reflects the dimly lit zone at the boundary region between the epipelagic and mesopelagic, and at BATS has been considered to reach from the base of the photic zone to ~300 m depending on depth sampling resolution, whereas the mesopelagic reaches 1,000 m (41, 46).

Hikarchaeal amplicons were present in 85% of the 2016–2021 profile samples (*n* = 602). They formed 0.001%–0.984% of total bacterial and archaeal (prokaryote) relative abundance, or 0.002%–3.134% of total archaeal amplicons, and were below detection primarily at 0 m, 40 m, and 80 m (Fig. 2B; Data set S1; Fig. S1). Their highest contributions were between 110 and 300 m (median relative abundance 0.1% of total prokaryotes), based on statistical comparison of data from <80 m (median 0.0%), 80–110 m (median 0.01%), and >110–300 m (*P* < 0.001). They reached ≥0.5% of total prokaryotes in 18 samples and formed ≥2% archaeal relative abundance in 16 samples, largely in the summer stratified period and below 160 m. These results indicate that in contrast to hypothesized growth-stimulating conditions for MGI connecting to ammonia fluxes (47, 48), as well as high abundances of Poseidoniales reported in the surface ocean (49), the Hikarchaeia may respond to semi-labile DOM exported and trapped below the thermocline upon stratification (38, 39, 41). While acknowledging that relative abundance patterns are inherently influenced by the presence and dynamics of other taxa, and that detection of low-abundance taxa is more sensitive to sequencing depth, our results identify Hikarchaeia as members of the conditional rare biosphere, with ephemeral low-level “blooms” (50).

Depth and seasonality patterns emerged for hikarchaeal relative abundances whether analyzed in the context of contributions to total prokaryotic amplicons (Fig. 2B) or total archaeal amplicons (Fig. S1). Periods of higher hikarchaeal relative abundances aligned with intense stratification in comparison to winter deep mixing when computed out of total archaea (chi-squared = 13.2, df = 3, *P* = 0.004; Dunn *post hoc* between winter and summer *Z* = −3.1, df = 1, *n*_SS_ = 253, *n*_DM_ = 152, *P* = 0.02) and out of total prokaryotes significance varied (chi-squared = 10.3, df = 3, *P* = 0.002; *post hoc* between winter and summer *Z* = −2.1, df = 1, *n*_SS_ = 253, *n*_DM_ = 152, *P* = 0.16). We then binned samples collected within ±10 m of the DCM depth for each cast as “DCM” for summer stratified samples. Those collected between 0 m and <10 m above the DCM were defined as upper euphotic (UE), whereas those from >10 m deeper than the DCM and ≤350 m were defined as twilight zone. Higher relative abundances occurred in the TZ (Fig. 2B), with a median of 0.17% (TZ, *n* = 152) Hikarchaeia relative abundance out of prokaryotes, versus 0.00% (UE, *n* = 85) and 0.01% (DCM, *n* = 16). Time-series depths from 500 to 1,000 m were collected from 2019 to 2021 only (*n* = 107). We defined the core mesopelagic as >350 m to ≤1,000 m, which exhibited a summer stratified hikarchaeal median of 0.03% out of prokaryotes and 0.06% out of archaea (*n* = 60).

**Fig 3:**
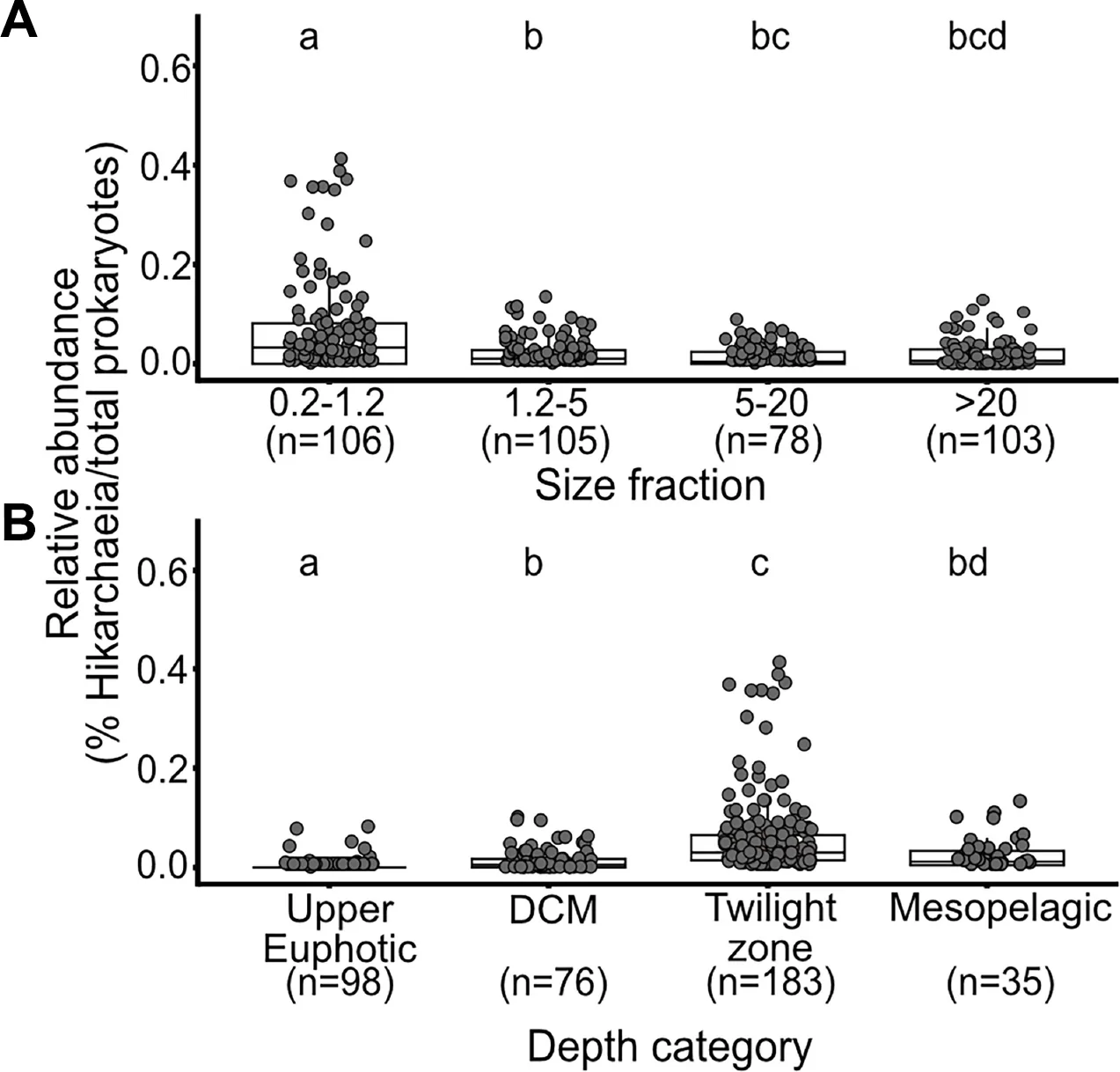
Hikarchaeia across size fractions and depth zones. (**A**) The mean, upper, and lower quantiles of (**A**) hikarchaeal relative abundances out of total prokaryotic amplicons in four size fraction groups 0.2–1.2 µm, 1.2–5 µm, 5–20 µm, and >20 µm sampled during pump casts in July 2018, July 2019, August 2021, and November 2021 at BATS (38). Letters indicate statistical differences between size fractions (χ^2^ = 29.2, df = 3; *P*_0.2–1.2 vs 1.2–5_ = 0.0009, *P*_0.2–1.2 vs 5–20_ = 0.0001, *P*_0.2–1.2 vs >20_ < 0.0001). (**B**) Hikarchaeal relative abundances out of total prokaryotic amplicons in the UE zone (between 0 m and <10 m above the DCM), DCM (10 m above and below the DCM, see reference 38), twilight zone (between >10 m below the DCM and ≤350 m), and core mesopelagic (between >350 m and ≤1,000 m). Comparisons were significant, χ^2^ = 172.5, df = 3; *P* ≤ 0.01, except for DCM versus core mesopelagic, which was *P* = 0.09. Individual data points are shown as dots, and the casts were performed at BATS as indicated in panel **A**.

We then examined Hikarchaeia distributions in the free-living (FL) fraction, operationally defined as the 0.2 to <1.2 µm size fraction, versus larger size fractions. Published sequentially size-fractionated large volume (50–370 L) *in situ* pump-cast V4 16S rRNA gene amplicons from BATS (39) were used that had been collected during the summer stratified period and autumn transition between 2018 and 2021 (Data set S2). Hikarchaeal relative abundances were significantly higher in the free-living (<1.2 µm) versus particle-associated (1.2–5 µm, 5–20 µm, >20 µm) size fractions both when considered out of total prokaryotes (Fig. 3A) and total archaea (Fig. S2). These results differ from trends reported for the entire archaeal community, where relative abundances of Archaea as a whole were highest in the 1.2–5 µm fraction, suggesting larger cell sizes or particle association (39). With respect to depth zones, here, we noted that the DCM varied between 69 and 134 m in the pump casts, and samples were binned by depth zones as in the above section (Fig. 3B). Statistical testing confirmed hikarchaeal mean relative abundances out of prokaryotes were highest in the TZ (0.06% ± 0.08%, *n* = 183) compared to the UE (0.003% ± 0.01%, n=98), DCM (0.02% ± 0.02%, *n* = 76) and mesopelagic (0.03% ± 0.03%, *n* = 35), and likewise out of archaea (Fig. S2B). Combined with the pronounced seasonality and depth-related trends in monthly profiles, this suggests that semi-labile sources of surface DOM, trapped below the seasonal thermoclines, either directly or indirectly stimulate Hikarchaeia.

### Hikarchaeal evolution involves divergence into five major lineages

To gain insights into the evolutionary history of Hikarchaeia, we generated metagenomes at 200 m during the stratified period, where higher relative abundances of Hikarchaeia had been observed (Fig. 2B, pink star). Metagenome assembly methods were optimized through repeat-sampling over ~70 hours to generate an initial six BATS metagenomes (Data set S3). Co-assembly resulted in a complete hikarchaeal genome (HikBATS200m) of 1.17 Mbp, with 1,194 predicted protein-encoding genes. In addition to being circularized, an indication of completeness, the sequence included a complete rRNA operon sequence and 43 tRNAs for codons of all 20 amino acids (Data set S4).

**Fig 4:**
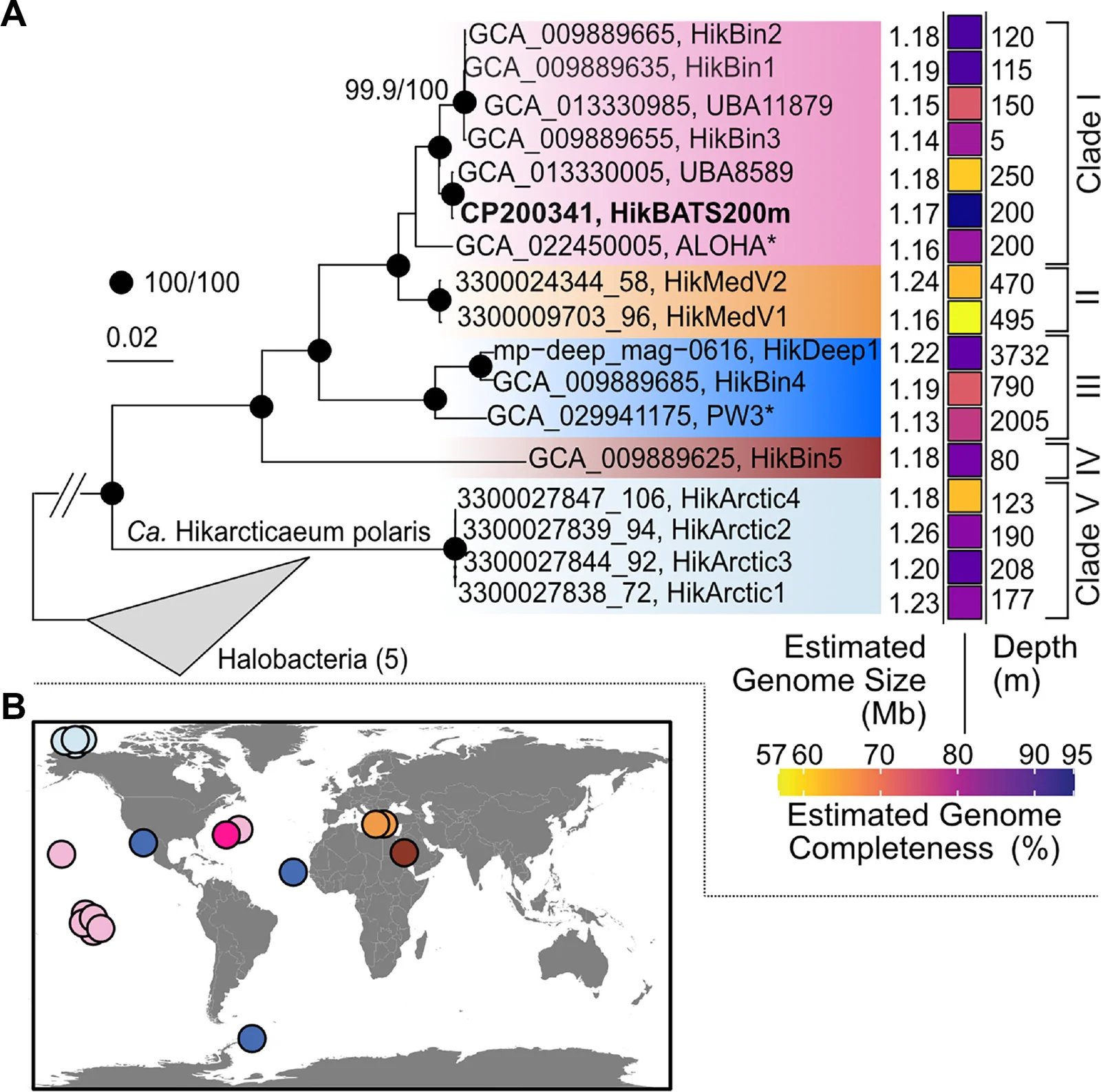
Evolutionary relationships and vertical distributions of Hikarchaeia clades. (**A**) Phylogenomic reconstruction based on 85 single-copy marker genes (SCMGs) recovered from high- (≥90% completeness and ≤5% contamination) and medium- (≥50% completeness and ≤10% contamination) quality MAGs (Data set S5). Bold text indicates the circularized genome from BATS (HikBATS200m). Bipartition support values depicted at nodes are based on SH-aLRT/UFbootstrap using 1,000 replicates; colors highlight statistically supported clades. The right-hand columns reflect estimated genome size (Mb), estimated MAG completeness (%), and depth (m) where recovered. (**B**) Geographic locations of samples from which metagenomes were assembled (colors as in panel **A**).

Previously reported hikarchaeal MAGs come from *Tara* Oceans data and are either medium (two) or high quality (three) (23). They were assembled from sampling in the Pacific Ocean at 5 m (HikBin3) and 115–120 m (HikBin1 and HikBin2), the Southern Ocean at 790 m (HikBin4), and the Red Sea at 80 m (HikBin5). Here, in addition to the BATS hikarchaeal genome, we identified four “new” hikarchaeal MAGs in published sequence data from the Canadian Basin (51), representing the first Arctic hikarchaeal MAGs (Data set S5). We identified seven other medium-quality hikarchaeal MAGs assembled from a range of habitats, two from near Aegean Sea submarine volcanoes, one from Station ALOHA in the North Pacific Subtropical Gyre (52), and one each from the North Atlantic (53), tropical Atlantic, tropical Pacific Ocean (31), and the Guaymas Basin (54), located in the Gulf of California (Fig. 4A and B). Collectively, the HikBATS200m genome, alongside other hikarchaeal MAGs, enabled new analyses of the evolutionary and functional attributes of Hikarchaeia.

Phylogenomic reconstruction differentiated Hikarchaeia from other members of the class Halobacteria, as expected (21, 23, 55). A published analysis of the five “HikBin” MAGs, based on 56 conserved proteins, led to a topology indicating three hikarchaeal groupings (23). Here, we delineated five statistically supported clades, named herein Clades I to V (Fig. 4A; Data set S5). Clade I contains HikBin1, HikBin2, and HikBin3, which were proposed to form the species *Candidatus* Hikarchaeum yamanae (23), and HikBATS200m. This clade contained MAGs assembled from photic and twilight zone samples. Our expanded taxonomic sampling identified Clade II, which contains MAGs from near a submarine volcano, as a new sister group to Clade I. Three singlet branches were present, and unlike that designated Clade IV (GCA_009889625, HikBin5), the MAG PW3 (within Clade III) and the ALOHA MAG (which lacked bootstrap support) were of lower quality, which interferes with phylogenetic inference. Hence, we did not differentiate these two singlets from other groupings. Additional higher-quality data will be needed to pursue their relationships. We also discovered a deep-branching hikarchaeal lineage, Clade V, comprising uncharacterized MAGs from the Arctic Canadian Basin (51). We propose that Clades I–IV capture species-level divergence (e.g., Hikarchaeaum Clade II), and in the case of Clade V, greater divergence. Thus, we also propose that the latter has at least genus-level divergence, if not family level, and name it genus species *Candidatus* Hikarcticaeum polaris.

### Linking phylogenomic analyses to rare biosphere biogeography

Prior studies have pointed to the low abundance of Hikarchaeia as a major factor behind poor understanding of this class (1–3), which currently has just a few existing MAGs, and only three that exceed 90% completeness. High population-level diversity can hinder metagenomic assembly (56). While data on hikarchaeal population diversity are lacking, their low relative abundances suggest that they are indeed part of the rare biosphere, contributing to difficulties in recovering MAGs. Because metagenomic read-mapping works poorly for examining environmental distributions of rare or microdiverse taxa in nature, we used another approach contextualized by knowledge from the phylogenomic analysis.

The hikarchaeal MAGs came from multiple habitats and depths (Fig. 4A and B; Data set S5), some corresponding to where MGIV 16S rRNA genes were initially discovered, specifically, deep ocean (28, 30) and Arctic (29) waters. Here, 16S rRNA genes from hikarchaeal MAGs were used to bring together phylogenomic clades and environmental clone library data, using nucleotide identity metrics and clade-specific 16S rRNA gene diagnostic positions (Data set S6 to S8). This was not possible for Clade IV because the HikBin5 MAG lacks a 16S rRNA gene sequence; additionally, Clade II only had one MAG with a 16S rRNA gene sequence, preventing delineation from Clade I. Clade I MAGs containing 16S rRNA sequences in their assemblies (HikBATS200m and HikBin1-3) had 100% 16S rRNA gene nucleotide identity. Clade III, comprising MAGs from >780 m depth, also contained the first MGIV 16S rRNA gene sequence reported, coming from 3,000 m Antarctic Polar Front, as well as 2,540 m (Pacific) and 1,503 m Guaymas Basin sequences (28, 30, 57). The original Arctic 16S rRNA gene sequence (29) grouped with Clade V. Collectively, the geographical distribution of these MAGs and their phylogenomic groupings pointed to possible habitat differentiation (58), which will require data from a greater number of locations to fully characterize.

**Fig 5:**
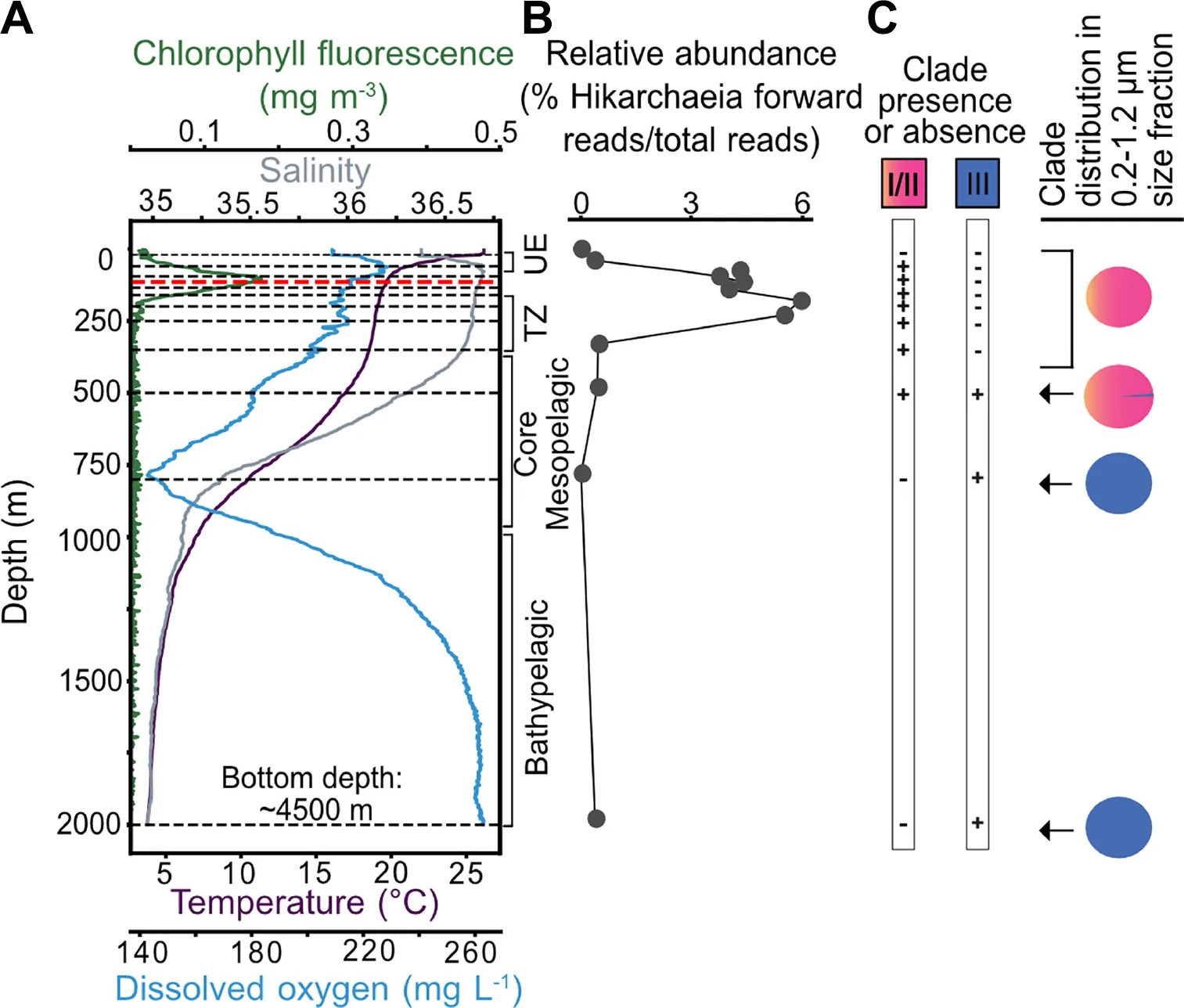
Hikarchaeal distributions from the surface to bathypelagic. (**A**) *In situ* profile extending from 20 to 2,000 m in July 2022 shows (left panel) variations in salinity, dissolved oxygen (mg L^−1^), chlorophyll (mg m^−3^), and temperature (°C). Note, we acknowledge the mesopelagic is defined as 200–1,000 m (45), and here delineate the core mesopelagic (>350 to ≤1,000 m) and TZ (>10 m below the DCM and ≤350 m). (**B**) Discrete depths sampled with *in situ* pumps are indicated by dashed lines (DCM in red); (middle panel) hikarchaeal relative abundance out of total reads in the FL size fraction (≥0.2 to <1.2 µm) where Hikarchaeia were most abundant. (**C**) “Plus” symbols indicate the clade was detected (“minus,” not detected). Proportions are indicated by pies, with pink/orange reflecting Clade I/II (presumably Clade I based on findings herein) and blue reflecting Clade III. Clade V-related sequences were not detected at BATS. The divergent Clade IV MAG (Red Sea) lacks 16S rRNA gene information, preventing searches.

We performed additional sampling to pursue hikarchaeal clade distributions deep into the BATS water column. Specifically, in 2022, we deployed large-volume pumps extending into the bathypelagic during the summer stratified period when seasonally resolved amplicon profile data indicated highest relative abundances (Fig. 2B and 5A; Data set S9). Relative abundances were higher in the TZ than in the UE, or at mesopelagic or bathypelagic depths (Fig. 5B). They were consistently higher in the free-living size fraction (Data set S9). Clade I/II dominated hikarchaeal relative abundances in samples from the surface to 500 m, whereas in the 800 m sample Clade III dominated and Clade I/II was no longer detected (Fig. 5C). Clade V amplicons were not detected at BATS.

### General attributes of Hikarchaeia

**Fig 6:**
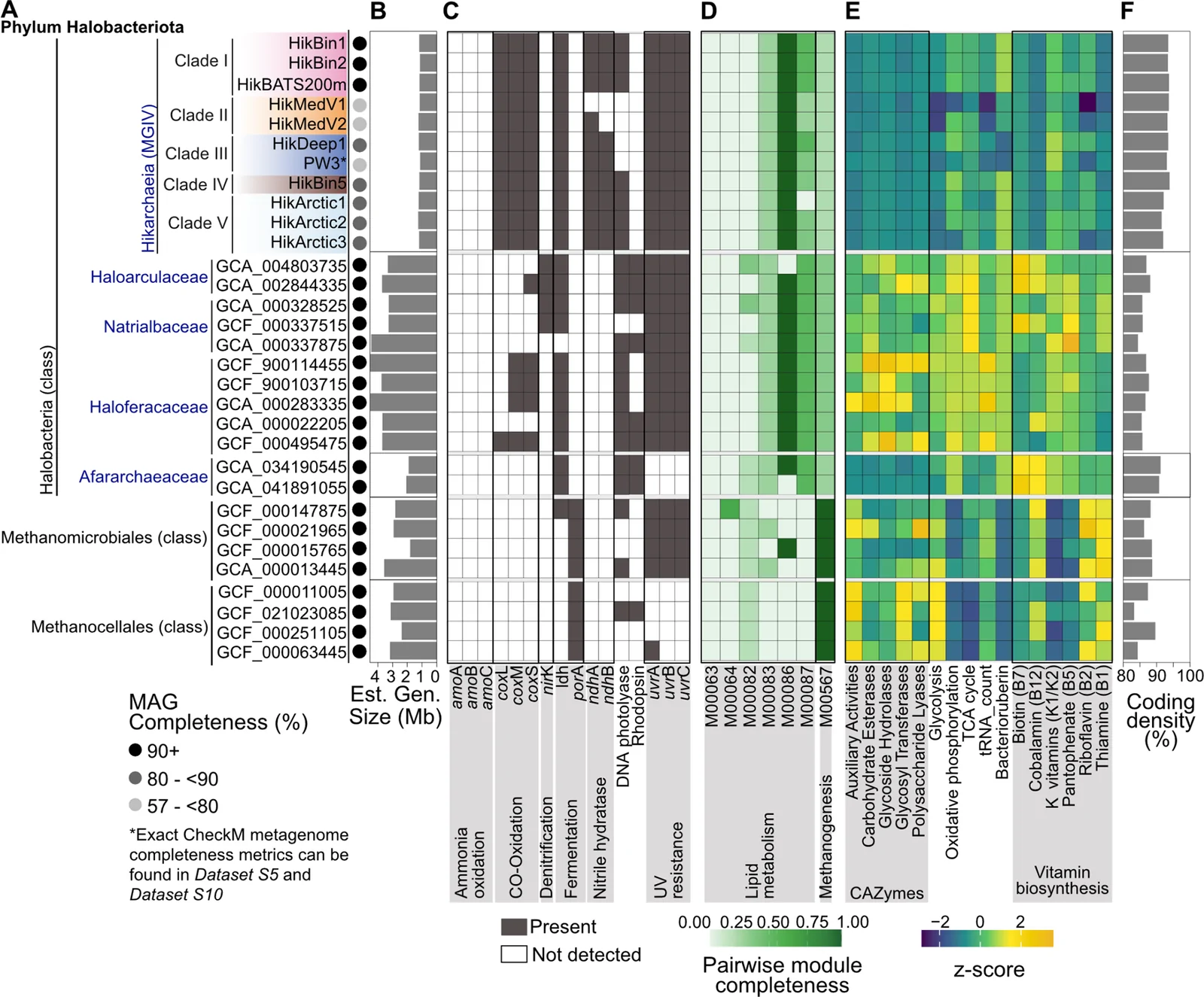
Genome characteristics of Hikarchaeia relative to select other lineages within the Halobacteriota phylum. (**A**) MAG identifiers with estimated completeness indicated by categories (circles) as defined in the panel. Current naming of taxonomic levels are indicated. Blue text indicates family-level designations within the order Halobacteriales. (**B**) Estimated genome size of MGIV clade representatives alongside reference genomes/MAGs (Data set S11). (**C**) Protein presence/absence patterns of marker genes associated with selected metabolic pathways and functions of interest in marine studies; note that not all MAGs used are high quality, and patterns could be influenced by incomplete MAG data (see panel **A**). (**D**) Pathway completeness for lipid metabolism and methanogenesis pathways, scored between 0 and 1, where 1 indicates 100% completion. (**E**) Enrichment of carbohydrate-active enzyme (CAZyme) classes and genes associated with carbon metabolism, respiration, and vitamin biosynthesis, scored according to deviation from the mean count (*Z*-score). (**F**) Protein-coding densities for the same taxa. Note that these relationships have been studied extensively in references 21, 23.

Relationships between the Hikarchaeia family and other members of the phylum Halobacteriota, including other Halobacteriales families, have established patterns of gene loss in this taxon (20, 21, 23, 55). We note that taxonomic level designations are in flux, with an originally described class-level designation as Hikarchaeia, and variation from there in GTDB, with this group now representing a family (Hikarchaeaceae) rather than a class. Regardless, most of these other lineages are not notable in pelagic marine environments. While our primary focus was ecological, we performed cursory comparisons to other classes in the Halobacteriota, and several families within the Halobacteriales, which include Hikarchaeia based on GTDB taxonomy (Fig. 6A through F). This confirmed the smaller genome sizes of Hikarchaeia (Fig. 6B) and higher coding density (Fig. 6F) than for the other Halobacteriales in our comparison, but similar lipid-encoding profiles (Fig. 6D). Gene loss (or less redundancy) within various biosynthetic pathways were generally greater than in other Halobacteriales families, apart perhaps from the Afararchaeaceae (Fig. 6E) (23). Evolutionary analyses through phylogenetic reconstructions and more comprehensive metabolic analyses are available in reference 23. With respect to proteins often discussed in the marine literature, we noted presumed loss of rhodopsin in Hikarchaeia (Fig. 6C). The presence of carbon monoxide (CO)-oxidation-related genes and nitrile hydratases discussed below, under functional specialization, may represent retention and gain of genes, respectively. Further evolutionary analyses are needed to establish the origins of these genes.

**Fig 7:**
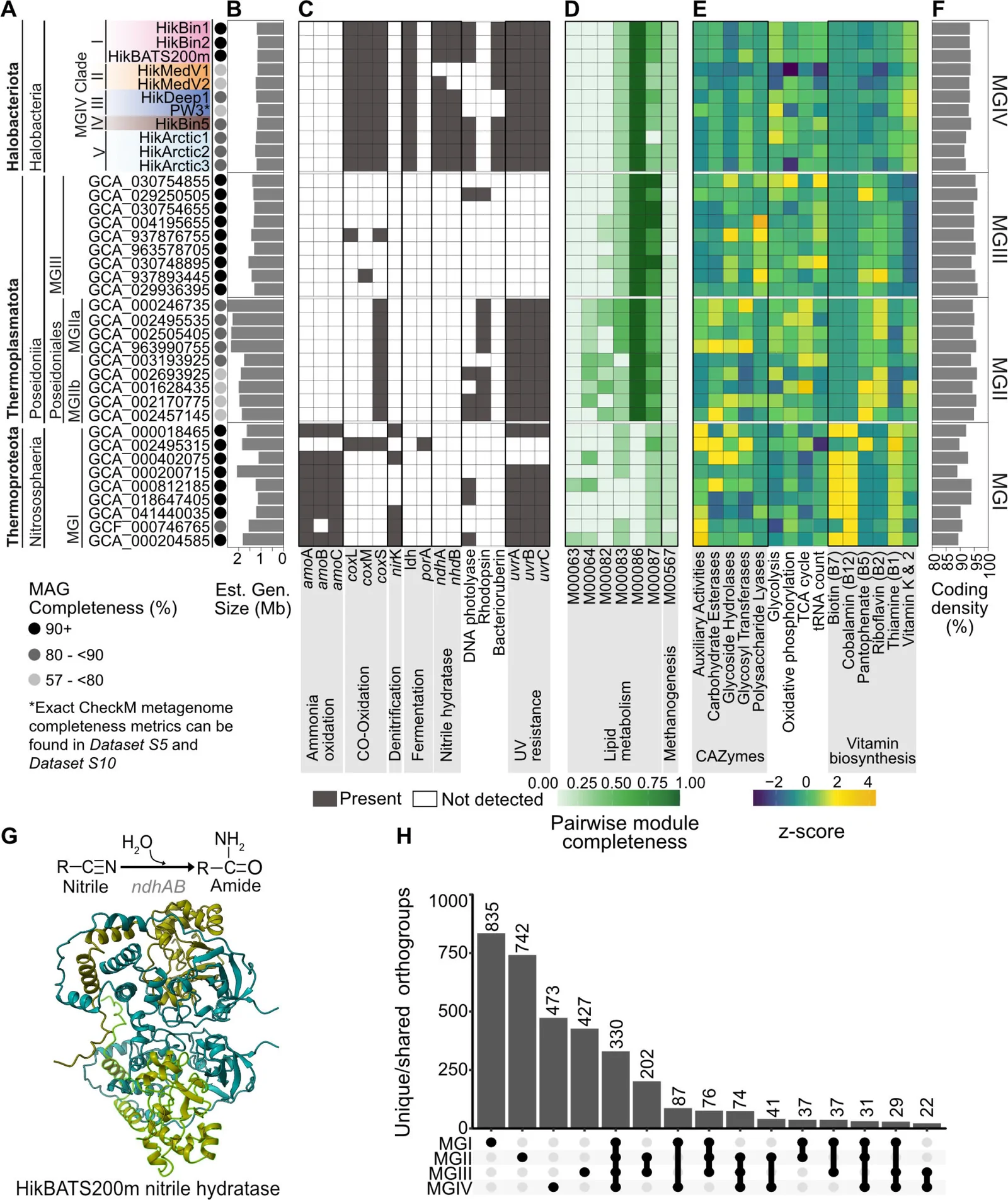
Genome characteristics of Hikarchaeia and other major pelagic marine archaeal groups. (**A**) MAG identifiers with estimated completeness indicated by categories (circles) as defined on panel. Current naming of phyla (bold), and taxonomic levels below are indicated to the left. (**B**) Estimated genome size of MGIV clade representatives alongside reference genomes/MAGs for Marine Groups I, II, and III (see Data set S10). (**C**) Protein presence/absence patterns of marker genes associated with selected metabolic pathways and functions of interest; note that not all MAGs used are high quality, and patterns could be influenced by incomplete MAG data (see panel** A** or Data set S11). (**D**) Pathway completeness for lipid metabolism and methanogenesis pathways, scored between 0 and 1, where 1 indicates 100% completion. (**E**) Enrichment of CAZyme classes and genes associated with carbon metabolism, respiration, and vitamin biosynthesis, scored according to deviation from the mean count (*Z*-score). (**F**) Protein-coding densities for the same taxa. (**G**) The reaction catalyzed by nitrile hydratase with a model of the nitrile hydratase protein below (based on prediction from the HikBATS200m protein). The α (olive) and β (teal) chains that form the heterodimers are indicated based on AlphaFold3 predicted template modeling and interface predicted template modeling scores of 0.88 and 0.86, respectively. (**H**) Patterns, including counts, of unique and shared protein orthogroups between MGI and IV, where a cutoff of detection in three MAGs was used to indicate presence in a group (for MGIV, it needed to span at least two clades).

Next, we studied predicted protein functions in the hikarchaeal MAGs in the context of other pelagic marine archaea. Thus, the Hikarchaeia, specifically Hikarchaeum Clades I–IV and Hikarcticaeum Clade V, were compared to MGI, MGII, and MGIII, by selecting a set of 9–10 marine-associated representatives for each group (Fig. 7; Data set S10). We aimed for the best quality MAGs available, by necessity some being MQ, so that average predicted MAG genome completeness was 96% ± 4% (MGI, *n* = 9), 77% ± 8% (MGII, *n* = 10), 91% ± 1% (MGIII, *n* = 9), and 82% ± 13% for Hikarchaeia (*n* = 11). Within Hikarchaeia, average estimated genome size for Clade I was 1.17 ± 0.02 Mbp (*n* = 7), akin to that of Clade IV (Data set S5) but smaller than Clade V (1.22 ± 0.03 Mbp, *n* = 4, *P* < 0.01). Hikarchaeia as a whole have a statistically smaller average predicted genome size (1.19 ± 0.04 Mbp, *n* = 17; ANOVA, df = 3, *P* < 0.002) in comparison to MGI (1.52 ± 0.35 Mbp, *n* = 9) and MGII (2.07 ± 0.26 Mbp, *n* = 10) but not MGIII (1.38 ± 0.09 Mbp, *n* = 9, Data sets S5 and S10; ANOVA, df = 3, *P* = 0.14). Hikarchaeia and MGI appeared to have lower protein-coding density, 93% ± 1% and 91% ± 2% of the genome, respectively, than MGII and MGIII, which both had 95% ± 1%. Halophilic taxa had lower coding density (87.09% ± 2.07%, *n* = 12) and substantially larger genome sizes (3.52 ± 0.85 Mbp, *n* = 12; ANOVA, df=3, P < 0.001; Fig. 6; Data set S5).

The major metabolic pathways predicted from hikarchaeal proteins align with prior analyses of functions encoded by HikBin1–5 (see e.g., Fig. 6 and 7) (23). Hikarchaeia were reported to be aerobic heterotrophs, encoding the Embden-Meyerhoff pathway (glycolysis), and harboring a TCA cycle although the key glucogenic enzyme fructose 1,6-bisphosphatase was reportedly lacking (23). Here, it was detected in all hikarchaeal MAGs analyzed (Data set S11A and B). MGII and MGIII are also predicted to be heterotrophs, unlike members from MGI which are chemoautotrophic or potentially mixotrophic (18, 59–62).

CAZyme distributions varied across groups in our analysis (Fig. 7E; Data set S12). Family diversity analyses were likely influenced by MAG completeness, and hence we looked primarily at the sum of families. Hikarchaeia encoded 11 ± 2 CAZyme families per MAG (*n* = 11), and MGI (14 ± 4, *n* = 9), MGII (15 ± 6, *n* = 10), and MGIII (12 ± 3, *n* = 9) were fairly similar. The fact that MGII and Hikarchaeia have lower predicted MAG completeness may bias results, since protein family diversity cannot be easily corrected for estimated genome size. Enrichment of specific families appeared to occur, e.g., MGI had the AA class, redox enzymes that support other carbohydrate-active enzymes. Likewise, MGII had CBM96, which is predicted to bind alginates, aligning with their abundance in surface waters during coastal phytoplankton blooms (26, 63). In MGIV, we found that glycoside hydrolases (GH), responsible for breaking down complex carbohydrates, were lacking except for the GH188 family, containing sulfoquinovosidases that hydrolyze glycosides and release the small molecule sulfoquinovose. The latter is an S-layer component in the extremophile archaeon *Sulfolobus* (64). With respect to overall numbers, Hikarchaeia have similar CAZyme numbers (20 ± 3, *n* = 11) as MGI, MGII, and MGIII when corrected for estimated MAG completeness. Low CAZyme numbers in Archaea relative to Bacteria have been reported previously for representatives of MGI, MGII, MGIII, and ‘Halobacterota’ (65). Halobacteriales that are purportedly ancestral to MGIV encode more CAZymes across more families, ranging from 40 to 151 CAZymes per genome, coming from 22 to 52 CAZyme families, based on the ten references examined herein (with Afararchaeaceae excluded, Data set S12). Notably, none of the hikarchaeal CAZymes are predicted to be localized extracellularly, while 8%, 23%, and 28% are for MGI, MGII, and MGIII, respectively. Hence, CAZymes encoded by Hikarchaeia appear to primarily serve in breaking down carbohydrates within the cell, not extracellular breakdown of carbohydrate-rich particulate organic matter.

### Functional specialization in Hikarchaeia

Proteorhodopsins are well known in MGII (59) and a subset of MGIII (18), as well as other marine microbes where they have predicted or established roles as proton pumps (66–68). We did not detect rhodopsin or enzymes for retinal pigment precursor steps (that are present in some rhodopsin-encoding marine prokaryotes) in Hikarchaeia; however, enzymes for sterol biosynthesis that lead into the retinal biosynthesis pathway were present, steps which overlap with other pathways. In HikBATS200m and most other hikarchaeal MAGs, a conserved set of eight genes were present, namely phytoene synthase (*crtB)* followed by phytoene desaturase (*crtI*), lycopene elongase (*ubiA*/*lyeJ*), and the carotenoid biosynthesis membrane protein (*cruF*, bisanhydrobacterioruberin hydratase), indicating capacity for *de novo* bacterioruberin biosynthesis (Fig. 7; Data set S11). Two bacterioruberin-synthesizing halophilic Halobacteria have just been reported with the same gene suite and, like Hikarchaeia, lack *crtD* present in model taxa like *Halorubrum* (69, 70). Bacterioruberin studies generally focus on biotechnological uses of halophilic Halobacteria, such as *Haloferax* and *Halorubrum*. In these, this long-chain C_50_ carotenoid has confirmed roles in membrane stability under high osmotic conditions, in protection from UV radiation, and as an antioxidant (70, 71). We are unaware of reports of the bacterioruberin pathway in other oceanic pelagic microbial taxa. The complete pathway was detected in Clades I, II, IV, and V. However, in Clade III’s HikDeep1 three conserved genes upstream (*sucC*, *sucD*, and *adh11*) of the pathway outlined above were directly followed by a partial *cruF*, and then by a conserved downstream gene (*bamB*). In another Clade III MAG, PW3.metabat2.160, the contig with the three genes identified upstream of the bacterioruberin pathway in other clades shows completely different genes following these three. This suggests that epipelagic and TZ lineages have retained the functional pathway from their halophilic halobacterial (putative) ancestors (Fig. 6), while the pathway is being lost by deep-dwelling Clade III members. Bacterioruberin’s role and influence on hikarchaeal cell physiology in their oceanic habitats will be exciting to elucidate.

Multiple regions of HikBATS200m encode xanthine oxidase family proteins, which include carbon monoxide dehydrogenase (72). The latter performs aerobic carbon monoxide oxidation, producing CO_2_ and reducing power. The three subunits for CO dehydrogenase, *coxM*, *coxS*, and *coxL*, are encoded contiguously. These individual enzymes were noted previously (23), with hypothesized alternative roles in aromatic compound breakdown due to fragmentation across MAG contigs. We noted *coxG*, one gene downstream from *coxMSL*, and the intervening gene encoding GTP cyclohydratase involved in the synthesis of CO pterin cofactors. CoxG is a lipid-binding protein that anchors CO dehydrogenase to the cytoplasmic membrane in *Afipia* (formerly *Oligotropha*) *carboxidovorans* (73). *A. carboxidovorans* is a non-marine bacterium that reportedly grows chemolithoautotrophically using CO, or heterotrophically (74). In HikBATS200m, the operon gene order followed *Thermogemmatispora carboxidivorans*, an experimentally confirmed microaerophilic CO-oxidizing bacterium. CO oxidation using these enzymes is known in Halobacteria (72), whereas anaerobic (archaeal) methanogens (75) use different CO dehydrogenases. In addition, we identified the menaquinone biosynthesis pathway (*menB-F*, and the specific *menA* and *menG* for vitamin K2 biosynthesis), a lipoquinone that is highly abundant in halophilic halobacterial membranes (76). Menaquinone is known for its role as a membranous redox mediator in aerobic and anaerobic respiratory electron transport chains (77). CO oxidation has recently been coupled with menaquinone extraction from the membrane (by *coxG*), contributing to an active electron transport chain through the reduction of menaquinone to menaquinol (72). Experimental validation is required for the divergent xanthine oxidase family proteins in Hikarchaeia. This will help establish CO as either a C1 carbon source, which seems unlikely given hikarchaeal enrichment in the TZ where DOM from exported surface material is available (41, 45), or for energy, refining understanding of their niche dimension.

Finally, we identified nitrile hydratases in Hikarchaeia that previously have not been reported and were not detected in MGI, MGII, or MGIII. These metalloenzymes are known to cleave nitriles, semi-labile compounds, to amides (Fig. 7G), and are known in some marine bacteria from the Actinobacteria, Cyanobacteria, and Firmicutes (78, 79) as well as some marine protists (80). Based on modeling of the HikBATS200m nitrile hydratase, it comprises α and β subunits that form a heterodimer that binds iron or cobalt atoms. These findings indicate that some nitrile hydratases observed in ocean metagenomes (81) may come from Hikarchaeia. Moreover, there is a long-standing mystery of recalcitrant amide-N accumulation in the dark ocean, a form of high molecular weight dissolved organic nitrogen resistant to biological degradation (40, 82). This can be produced by nitrile hydratase activity, which among common marine pelagic Archaea appears to be a unique attribute of Hikarchaeia.

Because functional analyses by default ignore the vast amount of genetic dark matter in uncultivated microbes, we also analyzed protein families as orthogroups, agnostic to the presence or absence of functional annotations. Despite MGI representing a different phylum, there were 330 shared orthogroups across the four archaeal “groups” (Fig. 7H). The number of unique orthogroups in Hikarchaeia as a whole (473), based on detection in at least three MAGs, was similar to that in MGIII representatives (427), but almost twofold less than those exclusive to MGI or MGII (Fig. 7H).

With only one complete hikarchaeal genome and few HQ MAGs, comparative functional analyses or even orthogroup analyses are challenging, especially as they relate to the newly identified clades and their differentiation. Each hikarchaeal clade did appear to possess unique protein families, with 23 in Clade I, 1 in Clade III, and 35 in Clade V. None were detected in Clade IV and Clade II, potentially as a result of the few MAGs representing these clades and their poor quality, which compromises ortholog/homolog detection. Overall, most proteins that appeared to be unique to individual clades were of unknown function. However, several unique orthogroups in Clade V were annotated as cold- and stress-related, such as cold shock domain-containing proteins (Data set S13). The latter are also found in the Antarctic archaeon *Methanogenium frigidum* and have hypothesized roles in tRNA stability in hyperthermophiles (83). These results align with proposed genus- or greater-level divergence of Hikarcticaeum, providing first hints at features underpinning niche differentiation within the Hikarchaeia. Proteins of these functions would support Clade V in polar conditions, where it was recovered by metagenomics (Fig. 4) and in environmental 16S rRNA gene data (29).

### Conclusions

The ocean twilight zone plays a major role in the efficiency of the biological carbon pump—responsible for fueling the deep sea and for sequestration of atmospheric carbon (84). Archaea studies at coastal sites and in the open ocean have established broad patterns for MGI and MGII, while acknowledging little is known about MGIII and MGIV. Our studies highlight seasonal patterns in MGIV relative abundances in the North Atlantic subtropical gyre, and occupation of different global habitats by the five clades revealed herein. As rare biosphere members, Hikarchaeia are likely stimulated by specific environmental perturbations and shifts in biogeochemical conditions driven for some clades by stratification (50). Our results point to streamlined genomes and a heterotrophic, free-living lifestyle, with specialization on small molecules and potential importance of the C50 carotenoid bacterioruberin. Finally, accumulation of amides in the dark ocean (40) may well connect to their nitrile hydratase activities, providing an unseen role of Hikarchaeia in the nitrogen cycle.

## MATERIALS AND METHODS

### Oceanographic sampling

Seawater collections were performed aboard the *R/V Atlantic Explorer* between March 2016 and March 2021. Sampling was conducted at BATS (31°40′N, 64°10′W; bottom depth ~4,500 m) using Niskin bottles attached to a CTD rosette. Samples for V4 16S rDNA amplicon sequencing were collected at 1–300 m (eight depths; 2016 and 2018), 1–500 m (nine depths; 2017), or 1–1,000 m (12 depths; 2019–2021) with the rosette Niskin. Biomass from 4 L of seawater was filtered (per depth) onto a 0.22 µm Sterivex (polyethersulfone membrane) filter and stored in sucrose lysis buffer as described in reference 41.

Large volume size-fractionated DNA samples for additional amplicon sequencing were collected using *in situ* pumps (McLane). In each case, >50 L seawater was filtered sequentially through a 20 µm pore size Nitex screen (with a 150 µm Nitex backing filter), a 5 µm polycarbonate filter, a 1.2 µm polyethersulfone filter, and a 0.2 µm polyethersulfone filter. In total, 396 samples from such size fractions were produced in a prior study (39) focusing on the photic zone through mesopelagic in July 2018, July 2019, August 2021, November 2021, and reanalyzed herein. We also performed a deeper McLane pump cast capturing 12 depths extending to 2,000 m in July 2022.

Metagenomic samples were collected at 200 m using the rosette-mounted Niskin bottles roughly every 6 hours over four consecutive days from 8 to 11 July 2019. In this case, 20 L of seawater was transferred from the Niskin bottles into 20 L acid-washed, polycarbonate bottles and filtered onto a 0.22 µm pore polyethersulfone 142 mm diameter filter via peristaltic pressure filtration. Each sample was flash-frozen and stored at −80°C.

### DNA extraction and sequencing

DNA from biomass collected on 0.22 µm filters from Niskin profiles was extracted and analyzed for 73 profiles (607 samples). Samples from both Niskin bottles and *in situ* pumps that were used for amplicon sequencing were extracted using a phenol-chloroform protocol (85). For amplicon sequencing, DNA was amplified with 16S rRNA gene V4 primers 515F (5′-GTGYCAGCMGCCGCGGTAA-3′) and 806R (5′-GGACTACNVGGGTWTCTAAT-3′) with custom adapters (86). Libraries were pooled in equimolar concentrations, purified using the Qiagen gel extraction kit, and sequenced using 2 × 250 pair-end lanes with a MiSeq Reagent Kit v.2 (Illumina). Overall, sequencing depth for standard profiles was 87,756 (median), 100,100 ± 76,893 (mean ± S.D.) with *n* = 602, after exclusion of five samples with fewer than 10,000 reads (Data set S1). Samples from the upper 300 m had a sequencing depth of 88,455 (median) and 103,265 ± 83,482 (mean ± S.D.) with *n* = 495. For data used from a prior pump-cast study (39), these numbers were 32,047 (median), 31,739 ± 13,531 (mean ± S.D.) with *n* = 392 (Data set S2), after excluding samples with below 10,000 reads. Note that sequencing in reference 39 was also performed using 2 × 250 pair-end lanes and Illumina MiSeq. For each pattern detected, we also tested whether there were statistical differences in sequencing depth (see “Visualization and statistics” below). For pump cast data shown in Fig. 5 (July 2022), sequencing depth was 54,508 (median), 55,328 ± 6055 (mean ± S.D.) with *n* = 12 (0.2–1.2 µm size fraction, Data set S3).

For metagenomic sequencing, DNA was extracted from a quarter of each 142 mm polycarbonate filter using the DNeasy PowerWater Kit (Qiagen), with alterations including increasing the elution buffer temperature to 65°C and incubating for 2 minutes before DNA elution. The DNA was purified using 1.5× SPRI beads (Beckman Coulter). Five hundred nanograms of DNA was used to generate each NovaSeq 6000 library (Illumina; manufacturer’s protocol). After amplification (one cycle for adapter dimer removal) and assessment of library quantity (Qubit, Thermo Fisher) and quality (Bioanalyzer, Agilent), library DNA was sequenced as 2 × 150 bp paired-end sequence reads on NovaSeq S4 (Illumina).

### V4 16S rRNA gene amplicon processing

Amplicons were trimmed and dereplicated, and chimeras were removed using the DADA2 R package, v.1.2 (87). The resulting amplicon sequence variants (ASVs) were taxonomically classified using the DADA2 R package, v.1.2 (87), and the SILVA database. All V4 16S rRNA gene forward and reverse raw data were screened for reads with at least 95% sequence similarity relative to Hikarchaeia 16S rRNA genes, which were recovered from medium- to high-quality MAGs considered herein (Data set S5 and S7) and the 16S rRNA genes from MGIV clones in references 28, 29, using NCBI BLAST (BLAST + 2.15.0). The total abundance of these identified Hikarchaeia sequences was then counted for each sample, creating a hikarchaeal forward and reverse read count. The total number of forward and reverse reads per sample was also calculated. The unsystematic difference in hikarchaeal read counts between the forward (higher counts) and reverse reads (lower counts), as well as the stringency of the denoiser program used by DADA2, led to the underestimation of Hikarchaeia in the DADA2 amplicon data. Therefore, only forward-read data were considered here, see also reference 88. Estimated counts and relative abundances of Hikarchaeia for each sample were calculated as follows: (i) retention factor = DADA2 total amplicon count/total forward read count, (ii) estimated Hikarchaeia count = retention factor × Hikarchaeia forward read count, and (iii) percent Hikarchaeia relative abundance = 100 × (estimated Hikarchaeia count/(total Archaea [or total prokaryote] DADA2 amplicon count − Hikarchaeia DADA2 amplicon count + estimated Hikarchaeia count). Samples with less than 10,000 total forward reads (i.e., five for time-series data and four from large-volume size-fractionated data) were excluded from further analysis, with a final sample count of 602 for the time series and 392 for the large-volume size-fractionated data. Finally, for samples with a total archaeal DADA2 amplicon count between 1 and 10, the estimated Hikarchaeia count was set to zero.

### Visualization and statistics

Figure generation and data exploration were done in R using tidyverse and ggplot2 (89, 90). Maps were plotted using the ncdf4, raster, and ggplot2 packages in R (91–93), with ocean temperature data obtained from NOAA Coastwatch at a blended 5 km resolution. The relative abundances of Hikarchaeia ASVs out of total prokaryotic ASVs and out of total archaeal ASVs were plotted across time and depths using the mba package for Multilevel B-spline Approximation in R (94). After testing for normality and equal variance, the statistical test used was Kruskal-Wallis. Note, we also performed statistical tests on sequencing depth between periods, showing statistical differences for hikarchaeal relative abundances or counts, and found no significant differences between seasons (in sequencing depth, chi-squared = 7.5, df = 3, *P* = 0.06) or between pump cast depth categories (chi-squared = 3.8, df = 3, *P* = 0.3). Between pump cast size fractions, nearly significant differences were observed (chi-squared = 7.8, df = 3, *P* = 0.05); however, a Dunn’s *post hoc* test indicated that there were no significant differences in sequencing depth between the size fractions. For metagenome statistics, ANOVA with a Tukey HSD *post hoc* was used to compare differences.

### Metagenome assembly and binning

All sequenced Illumina reads were trimmed and quality controlled using Trimmomatic (v.0.39 [95]) with settings: “ILLUMINACLIP:TruSeq3-PE.fa:2:30:10 LEADING:3 TRAILING:3 SLIDINGWINDOW:25:30 MINLEN:50.” Trimmed reads were separately assembled for each sample using MEGAHIT (v.1.2.9 [96]) with a minimum kmer multiplicity of 1 and kmers set to 21, 29, 39, 49, …, 129, 141. Assembled contigs shorter than 5 kb were subsequently removed using the anvi’o (v.8 [97]) script “anvi-script-reformat-fasta.” Trimmed reads from all samples were mapped to filtered contigs from all samples separately with bowtie2 (v.2.4.2 [98]) using only paired reads and removing unaligned reads from the sequence alignment map. Sequence alignment maps were converted to binary format using the anvi’o script “anvi-init-bam” and binary alignment maps for the same sample were converted to one-depth profiles for differential coverage binning using the “jgi_summarize_bam_contig_depths” script from metabat2 (v.2.12.1 [99]). Assembled metagenomes were binned using the depth profile and previously filtered contigs in metabat2 with default settings.

Bin quality was estimated using checkM (v.1.1.3 [100]) running the subcommands “tree,” “lineage_set,” “analyze,” and “qa,” all with default settings. Bins were screened for (partial and complete) ribosomal rRNA using the checkM utility script “ssu_finder” with “-e 1e-05” as the significance cutoff. Bin taxonomy was predicted using GTDB-Tk (v.1.4.1 [101]) running “gtdbtk identify” followed by “gtdbtk align” and “gtdbtk classify” with default settings. Proteins were predicted using Prodigal (v.2.6.3 [102]) with default settings and subsequently annotated with DIAMOND blastp (v.0.9.14.115 [103]) against the NCBI nr database (downloaded 2021-06-15) with “--evalue 1e-04” and “--max-target-seqs 10” as cutoffs. Additionally, proteins were annotated with eggNOG (v.2.0.1-14 [104]) using “-m diamond.”

### Manual Hikarchaeia assembly refinement to circularization

Independent binning of six metagenomic assemblies included six bins predicted to have >50% completeness and <10% contamination (referred to as medium- or high-quality bins as defined in reference 105; Data set S5) assigned to the same family of Halobacteria (f_UBA12382) based on GTDB-Tk. DNA GC content for these ranged between 41.61% and 42.00% (Data set S5), and each bin contained between 4 and 18 contigs, enabling manual refinement of MAGs. Contigs were merged based on 99% identity and overlap of at least 100 base pairs using Geneious (v.2021.0.3 [106]). Paired reads from the six metagenomic samples containing f_UBA12382 bins were mapped with bowtie2 against every contig merged to guarantee even coverage and support through paired reads overlapping the merged region. Overlapping regions confirmed as continuous through this approach were accepted as correctly merged contigs. This process was repeated iteratively until only one contig remained, which had overlapping ends, indicating a circular genome assembly. The contig was cut in half and remerged at the overlapping ends; reads were then mapped to the contig again to check for continuous and even coverage in this region, confirming circularity. This resulted in one circularized genome, here termed HikBATS200m; the (single) rRNA gene sequence was identified using checkM “ssu_finder,” as described above. Median coverage computed through mapping of reads from the six metagenomes used to assemble the genome using bowtie2 (as described above) ranged between 26.9 and 57.9 rpkm (reads per kilobase per million mapped reads). The presence of tRNAs, encoding codons for all 20 amino acids, was confirmed using tRNAscan-SE (107), and note that this analysis was done for the additional 16 Hikarchaeia genomes below.

### Phylogenomic reconstruction of hikarchaeal diversification

Phylogenomic reconstruction is based on 17 Hikarchaeia genomes (56.73%–95.14% completeness and 0%–2.26% contamination) and an outgroup of five members of related Halobacteria families (Data set S5) and was performed as previously described in reference 108. Briefly, SCMGs were identified using the prebuilt Halobacteria-specific ortholog data set (taxID: 83963) of the eggNOG database (v5.0 [104]) with hmmscan (v3.3.2; E-value <1 × 10^−30^ [109]). Orthologs with a single copy present in more than 95% of the genomes were filtered to guarantee that all proteins were unique and then aligned with MAFFT (v7.520; --auto [110]) and trimmed with trimAl (v1.4.rev15; “--gappyout” [111]) for single-gene tree construction with IQ-TREE (v2.3.4; options: -B 1000 -bnni -m MFP [112]). Potential SCMGs were subsequently assessed and filtered, and then re-aligned and concatenated using custom scripts as described in reference 113. A final set of 85 SCMGs resulted in a trimmed alignment with 16,123 amino acid positions. The final phylogenomic tree was then reconstructed in IQ-TREE with 1,000 ultra-fast bootstrap and SH-ALRT iterations using the LG + F + I + R3 model selected by ModelFinder (-m MFP). The phylogeny was visualized in R using the ggtree package (v3.6.1 [114]).

### Comparative genomics, functional analysis, and protein structural predictions

Pairwise average amino acid identity (AAI) between Hikarchaeia predicted proteins (Data set S14) was calculated using the aai.rb script of the “ecogenomics” collection (115) with default settings except for “-n 20.” Proteins were clustered into orthogroups through OrthoFinder (v2.5.5 [116]) set to perform homology searches through DIAMOND blastp with multiple sequence alignments and tree reconstruction with MAFFT and IQ-TREE. Paralog sharing between Hikarchaeia clades was calculated based on ortholog gene count output, not including genes unique to a single assembly. Detection was required in at least three genomes of each MG compared (i.e., to indicate presence between MGI and IV), and in two MAGs/genomes of each MGIV clade compared to establish orthogroup presence. Counts were visualized in an upset plot using ggplot2 (93) and the ggupset extension library. Functional analysis was performed using Anvi’o (v8 [97]) as follows: first, genomes were imported into Anvi’o using “anvi-gen-contigs-database,” followed by KEGG and KOfam annotation through “anvi-run-kegg-kofams.” Contig databases were then combined into a genome storage database by “anvi-gen-genomes-storage,” followed by creation of the final functional profile by “anvi-script-gen-function-matrix-across-genomes” with “--annotation-source” set to “KOfam” and repeated with “KEGG” in consecutive runs of the script. The list of metabolisms searched can be found in Data set S11. CAZymes were annotated using dbCAN v13 HMM profiles (based on CAZY db [117]) using hmmscan (v3.4) and filtered based on profile-specific e-value cutoffs ranging from <1 × 10^−3^ to <1 × 10^−5^ using the dbCAN hmmscan-parser.sh script. Only the best CAZyme annotation for each ORF was kept in subsequent analysis.

For functional comparison of marine Archaea clades, PFAM domains were annotated using hmmscan with E-value <1 × 10^−10^, CAZymes were annotated as described above, and KEGG orthologs were identified using the ghostkoala webserver (https://www.kegg.jp/ghostkoala/) December 2024 version. tRNAs for representatives of clades MGI–IV were identified using ARAGORN (118) with default settings. Finally, the structural model of the HikBin200m (Fig. 7G) nitrile hydratase (alpha subunit [*nth*A]: PF02979; beta subunit [*nth*B]: PF02211) was visualized using ChimeraX v1.9.

## Data Availability

Whole water V4 16S rRNA gene amplicon sequencing raw data are available through NCBI BioProject PRJNA769790. Samples were deposited in SRA under the accessions SRR31399127–SRR31399359 and SRR31627169–SRR31627557. Previously published size-fractionated V4 16S rRNA gene amplicon sequencing data were obtained through NCBI BioProject PRJNA1066814, and 2022 data were deposited under PRJNA1354501. The circularized Hikarchaeia genome assembly was deposited in NCBI BioProject PRJNA1218439 under accession CP200341. It reflects a co-assembly of metagenomes deposited as SAMN36770476, SAMN36770478, SAMN36770480, SAMN36770482, SAMN36770484, and SAMN36770486. Other relevant data can be found in DOIs 10.6084/m9.figshare.c.7868135, 10.26008/1912/bco-dmo.917255.7, 10.6084/m9.figshare.32579706, and 10.6084/m9.figshare.32582337.

## SUPPLEMENTAL MATERIAL

Supplemental figures mbio.01753-26-s0001.pdf Figures S1 and S2.

Supplemental data sets mbio.01753-26-s0002.xlsx Data sets S1–S14.

ASM does not own the copyrights to Supplemental Material that may be linked to, or accessed through, an article. The authors have granted ASM a non-exclusive, world-wide license to publish the Supplemental Material files. Please contact the corresponding author directly for reuse.
