# Supplementary material for: Seasonal and evolutionary patterns of planktonic Hikarchaeia reveal ecological niches of marine rare biosphere constituents
Source: mBio. 2026 Oct 19;17(11):e01753-26. doi: 10.1128/mbio.01753-26 (PMC13753752; doi:10.1128/mbio.01753-26)
Supplement: Supplementary file 1 [file mbio.01753-26-s0001.pdf]

Seasonal and evolutionary patterns of planktonic Hikarchaeia reveal ecological niches of marine rare biosphere constituents

Fabian Wittmers[1,‡], Nicole R. Dames[1,‡,*], Michelle Michelsen[2], Rachele Spezzano[1,4], Jacqueline Comstock[1,3], Kristin Bergauer[5], Kevin Vergin[6], Shuting Liu[7], Rachel Parsons[8], Ben Temperton[2], Mak A. Saito[9], Craig A. Carlson[3,8] and Alexandra Z. Worden[1,4,*]

Supplementary Fig. S1 and S2

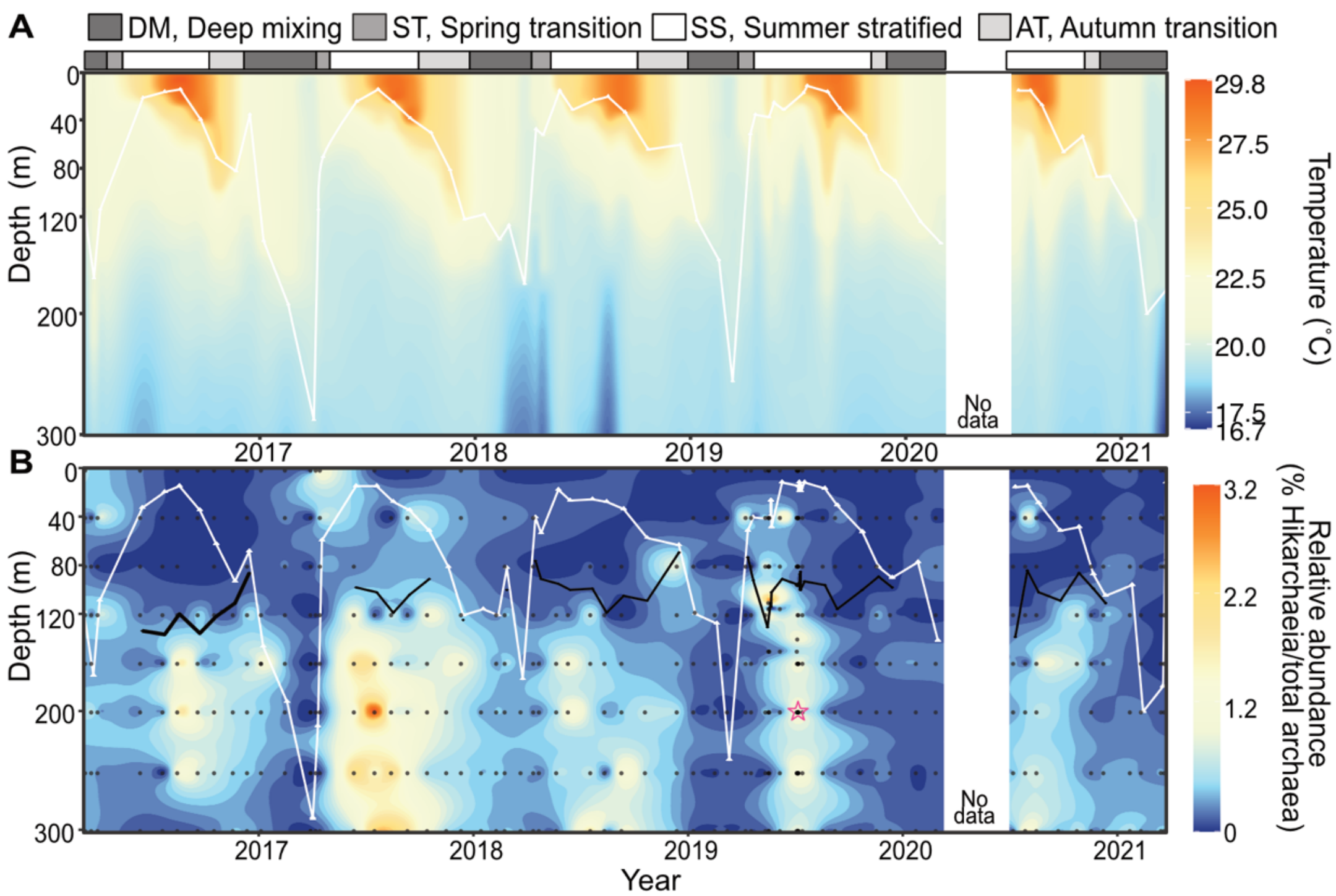

FIG S1 Seasonal dynamics and distribution of Hikarchaeia at BATS. (A) Temperature (ºC) over the upper 300 m of the BATS water column based on CTD data. Water column stability periods (above plot) are based on physical characteristics of the water column. Computed seasonal mixed layer depths (white triangles) are indicated. (B) Relative amplicon abundance of Hikarchaeia out of total archaea based on V4 16S rRNA gene data (*Dataset S1*) with interpolation from discrete samples (black dots, n=495). Seasonal mixed layer depth (white triangles) and Deep Chlorophyll Maximum (DCM; black diamonds) is also indicated when present. The star shows the depth and date of the 6 metagenomes used to assemble the HikBATS200m MAG (*Dataset S3*).

Periods of higher hikarchaeal relative abundances aligned with intense stratification in comparison to winter deep mixing (Chi-squared=10.06, df=3, nSS=313, nDM=189, p=0.02). We then binned samples collected within ±10 m of the DCM depth for each cast as "DCM" for summer stratified samples. Those collected between 0 m and <10 m above the DCM were defined as upper euphotic (UE), whereas those from >10 m deeper than the DCM and ≤350 m were defined as twilight zone (TZ). Higher relative abundances occurred in the TZ (Fig. 2B), with a median of 0.52% (TZ, n=152) Hikarchaeia relative abundance out of Archaea, versus 0.00% (UE, n=85) and 0.05% (DCM, n=16). Time-series depths from 500 to 1000 m were collected from 2019 to 2021 only (n=107). We defined the core mesopelagic (ME) as >350 m to ≤1000 m which exhibited a summer stratified hikarchaeal median of 0.06% (n=60).

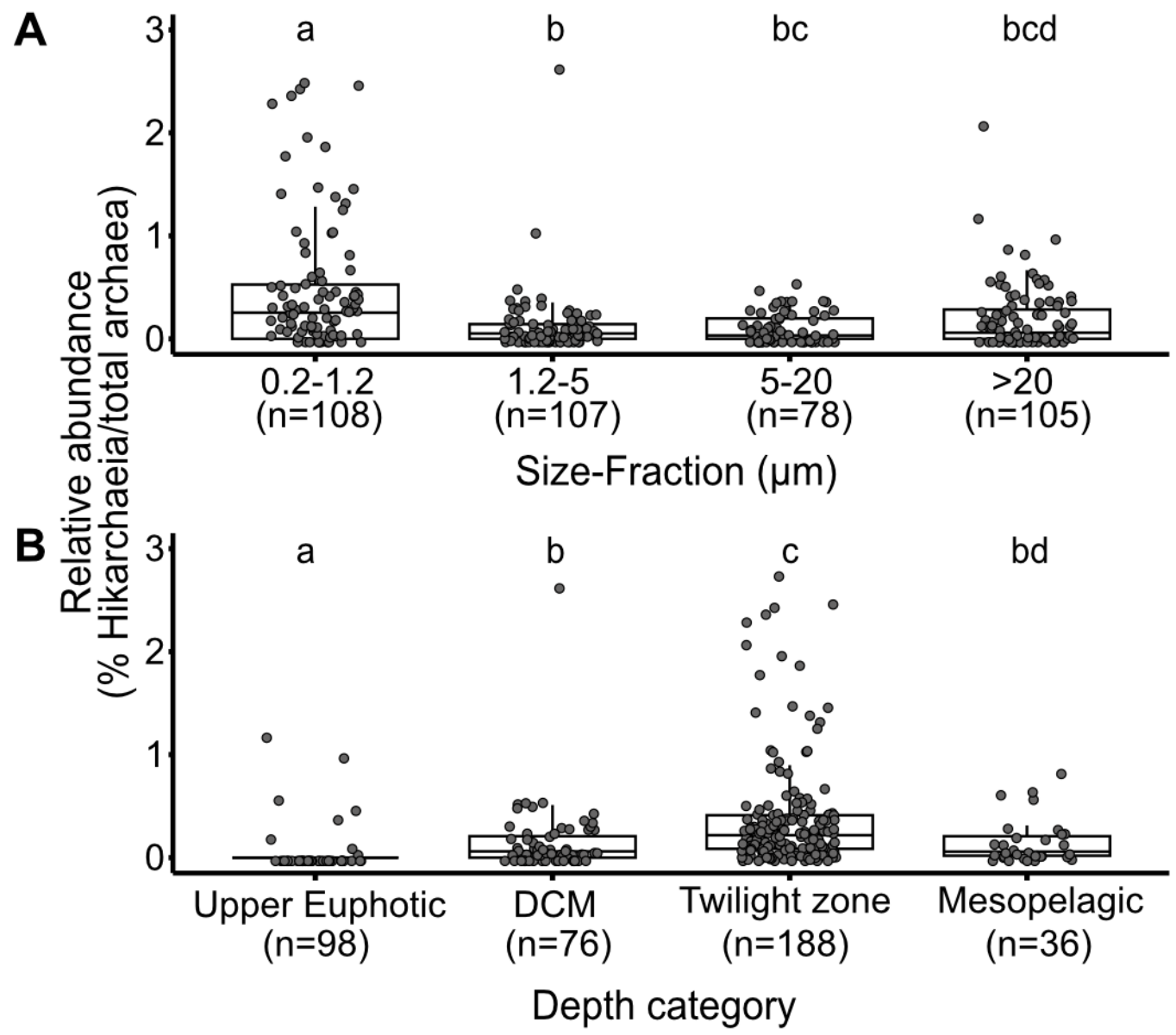

FIG S2 Hikarchaeia across size fractions and depth zones (computed out of total archaea). (A) The mean, upper and lower quantiles of (A) hikarchaeal relative abundances out of total archaeal amplicons in four size fraction groups 0.2-1.2 µm, 1.2-5 µm, 5-20 µm and >20 µm sampled during pump casts in July 2018, July 2019, August 2021, and November 2021 at BATS (38). Letters indicate statistical differences between size-fractions (χ^2=30.4, df=3; $p_{0.2\text{-}1.2vs.1.2\text{-}5}$<0.0001, $p_{0.2\text{-}1.2vs.5\text{-}20}$<0.0001, $p_{0.2\text{-}1.2vs.>20}$=0.0002). (B) Hikarchaeal relative abundances out of total archaeal amplicons in the upper euphotic zone (UE; between 0 m and <10 m above the DCM), DCM (10 m above and below the DCM, see (38)), twilight zone (between >10 m below the DCM and ≤350 m), and core mesopelagic (between >350 m and ≤1000m). All comparisons were significant, χ^2=157.2, df=3; p<0.0005. Individual data points are shown as dots, and the casts were performed at BATS as indicated in panel A.
